# Highly pathogenic avian H5N8 influenza viruses: should we be concerned?

**DOI:** 10.1080/21505594.2017.1386832

**Published:** 2017-11-27

**Authors:** M. D. Tate

**Affiliations:** a Centre for Innate Immunity and Infectious Diseases, Hudson Institute of Medical Research, Clayton, Victoria, Australia; b Department of Molecular and Translational Sciences, Monash University, Clayton, Victoria, Australia

**Keywords:** avian influenza, influenza virus, pathogenesis

## Abstract

Avian influenza A viruses pose a constant threat to global human health as sporadic infections continue to occur with associated high mortality rates. To date, a number of avian influenza virus subtypes have infected humans, including H5N1, H7N9, H9N2 and H7N7. The majority of ‘bird flu’ cases are thought to have arisen from direct contact with infected poultry, particularly in live markets in Asia.^1^ While human cases of the H5N8 subtype have not been documented as yet, there is the potential that H5N8 viruses could acquire mutations which favour infection of human cells. There is also the possibility that novel viruses with a tropism for human cells could be generated if H5N8 should reassasort with other circulating avian viruses, such as those of the H5N1 subtype. The emergence of a novel H5N8 virus with the capability of infecting humans could have drastic consequences to global health.

In the past, highly pathogenic avian influenza (HPAI) viruses have not been identified in typical wild bird reservoirs, limiting their prevalence by geography. However, outbreaks have occurred, as follows.^1^ In 1996 a HPAI H5N1 virus was detected Guangdong, China (clade 2.3.4), resulting in the death of wild birds. The virus then spread to over 80 countries, possibly via migratory bird paths. Clade 2.3.4 subsequently evolved, generating subclade 2.3.4.4 that includes subtypes H5N2, H5N5, and H5N8, all isolated in domestic ducks and poultry markets in China since 2010.^1,2^ HPAI 2.3.4.4 viruses subsequently spread to South Korea in 2014 causing an outbreak in domestic ducks and migratory birds.^3^ To date, isolates of H5N8 have been identified in North America, Africa, Europe and Russia and continue to cause avian outbreaks. The extensive distribution of HPAI H5N8, as well as the gene reassortment with other circulating avian viruses already observed for H5N8,^1,2^ suggests there is a potential risk for human cases of H5N8 infections.

In this issue of Virulence, Park *et al* performed a study to gain a greater understanding of the pathogenicity of H5 viruses.^4^ A panel of reassortant viruses compromising a H5N8 backbone (clade 2.3.4.4) with individual gene substitutions from a HPAI H5N1 virus (clade 2.2) were generated. The ability of the viruses to cause disease in mice and ferrets was examined. As previously reported,^5,6^ wildtype H5N8 showed moderate virulence for mice, in comparison to the parental H5N1 (80% and 0%, survival respectively). Substitution of the H5N8 PB2 with that of the H5N1 had the greatest impact on pathogenicity, which was associated with greater viral replication/organ spread, immunopathology and 100% mortality. The PB2 reassortant also displayed enhanced viral replication and pathogenicity in the ferret model, however, no aerosol transmission between individual animals was observed. Substitution of the PB2 gene also resulted in increased polymerase activity and replication in mammalian cells *in vitro*. These data suggest that if a novel H5N8 virus were to arise following substitution of the PB2 with a co-circulating virus, it could have a dramatic effect on pathogenicity but not necessarily transmission. Of note, substitution of the NA also resulted in 100% mortality and increased pro-inflammatory cytokine production; however, viral replication profiles were not significantly altered. PA, HA, NP, PB1, NS, M substitution reassortants demonstrated similar or attenuated virulence in comparison to the wildtype H5N8 virus.

In general, PB2 derived from avian viruses has a glutamate (E) at residue 627, whereas lysine (K) predominates in mammalian viruses.^7^ It has recently been reported that adaptation of H5N8 to mouse lung by serial passage results in the PB2 E627K mutation, as well as a HA A149V mutation.^5^ A reverse genetic approach was also utilised by Wu *et al* to demonstrate that the PB2 E627K mutation in H5N8 enhances polymerase activity and replication *in vitro* in mammalian cells. Interestingly, the H5N1 parental PB2, which its presence rendered the H5N8 reassortant highly virulent for mice in the study by Park *et al*,^4^ contains also a lysine (K) at position 627.^4^ It would therefore be of importance to examine the impact of substitution of the PB2 from other H5N1 viruses, such as those with a glutamate at 627. In an additional study, mouse-adaptation of H5N8 was also found to commonly result in the PB2 E627K mutation,^6^ although other mutations in the PA, PB1, HA, and NP were also observed. Of note, the mutation D701N in PB2 was also detected, which has been previously shown to enhance viral replication in mammalian cells.^8,9^

Circulating H5N8 viruses could, over time, obtain mutations which allow adaption to growth in human lung cells. A recent study has suggested that HPAI H5N8 viruses show an evolutionary trend towards gaining signatures present in human viruses.^10^ In the study by Xu *et al*, the genomes of H5N8 strains were analysed for conserved amino acids that allowed differentiation between avian and human viruses, termed species-associated signatures. Two human-like signatures (HLS) were identified in the PB2 gene, namely PB2-613I and PB2-702R. Interestingly, the 2 HLS were not detected in viruses before the 2014 outbreak, but were found in 80% of the analysed H5N8 viruses thereafter. The impact of these mutations on H5N8 pathogenicity is currently not known, but PB2-702R is adjacent to the characterised D701N mutation, which promotes growth of other subtypes in mammalian cells, as noted above.^8,9^

Collectively, these studies highlight the need for extensive surveillance of HPAI H5N8 viruses in poultry and wild birds. Although cases of human H5N8 virus infections have not been documented, the study by Park *et al* demonstrates that a single gene substitution could significantly enhance their pathogenicity in mammals.^4^ The possibility that a H5N8 virus could infect humans in the future can therefore not be ruled out. Additional studies are required to gain a greater understand the pathogenicity of H5N8 viruses in birds and mammals, as well as their potential ability to reassasort and/or adapt to humans. Lastly, the development of effective treatments for patients who present to hospital with severe ‘bird flu’ remains a priority.
